# Chains of carbon atoms: A vision or a new nanomaterial?

**DOI:** 10.3762/bjnano.6.58

**Published:** 2015-02-25

**Authors:** Florian Banhart

**Affiliations:** 1 Institut de Physique et Chimie des Matériaux de Strasbourg, UMR 7504 CNRS, University of Strasbourg, 23 rue du Loess, 67034 Strasbourg, France

**Keywords:** atom chains, carbon, carbyne, one-dimensional solids, sp^1^ hybridization

## Abstract

Linear strings of sp^1^-hybridized carbon atoms are considered as a possible phase of carbon since decades. Whereas the debate about the stability of the corresponding bulk phase carbyne continues until today, the existence of isolated chains of carbon atoms has meanwhile been corroborated experimentally. Since graphene, as the two-dimensional sp^2^-bonded allotrope of carbon, has become a vast field, the question about the importance of one-dimensional carbon became of renewed interest. The present article gives an overview of the work that has been carried out on chains of carbon atoms in the past one or two decades. The review concentrates on isolated chains of carbon atoms and summarizes the experimental observations to date. While the experimental information is still very limited, many calculations of the physical and chemical properties have been published in the past years. Some of the most important theoretical studies and their importance in the present experimental situation are reviewed.

## Introduction

Carbon is a unique element due to its ability to form bonds in three different hybridizations. The hybridizations, sp^1^, sp^2^, and sp^3^, correspond to bonding geometries in one-, two-, and three-dimensional space. Accordingly, the modifications of carbon have completely different appearance. The sp^2^- and sp^3^-bonded modifications, graphite and diamond, are known since ancient times, however it took until the beginning of the 21^st^ century until the synthesis and investigation of graphene, as the two-dimensional modification of carbon, became possible. Graphene has meanwhile evolved into a field of unprecedented activity in physics, chemistry, and materials research. Many of the unique and most interesting properties of graphene were found to originate from its two-dimensional nature. While extensive knowledge exists about the two- and three-dimensional allotropes of carbon, the sp^1^-hybridized one-dimensional modification [1] remains elusive. Although carbon chains have already been proposed by Tammann in 1921 [2], it is still not clear if sp^1^-hybridized carbon in a pure bulk form is chemically stable [3–5]. It is certain, however, that it is very reactive and it is possible that it is even explosive [6]. Some effort has been devoted to the synthesis and investigation of this phase, called carbyne or linear acetylenic carbon [7–8]. Attempts have been undertaken to synthesize this modification by using chemical techniques that stabilize the ends of the chains with nonreactive groups [9–11]. The synthesis of carbyne in its pure form, i.e., without end groups, turned out to be much more challenging and has been carried out, e.g., by cluster beam deposition [12–13].

It has even been proposed that carbyne could be the stable phase of carbon in a certain temperature-pressure regime, leading to its appearance in the phase diagram of carbon at very high temperatures [3]. Indications for natural carbynes have been reported in shock-compressed graphite in an impact crater [14], in meteorites [15], and by spectroscopic information from interstellar molecules [16–17]. However, the debate about the existence of carbyne at ambient conditions continues until today [6]. Nevertheless, it is undisputed that carbyne represents an exceptional state among the other modifications of carbon and will, most likely, never become available as a standard bulk material like graphite or diamond.

In the same way as graphite is composed of stacked sheets of graphene, carbyne is considered as an arrangement of linear chains of carbon atoms. The chains are therefore the elementary building blocks of sp^1^-hybridized carbon and the only real one-dimensional carbon phase (carbon nanotubes have often been denoted as one-dimensional carbon, however, they are cylinders and their properties depend strongly on the atomic arrangement in the two other dimensions). Like graphene sheets that can be rolled up in cylinders to form nanotubes, chains might also close their ends by forming atomic rings [18]. It has been recognized that chains of carbon atoms are not only remarkable (macro-)molecules; they may also have most interesting properties [19]. Two isomeric bonding types, involving either alternating single-triple bonds or only double bonds, lead to a variety of electrical properties, ranging from semiconducting to metallic. Furthermore, the strong covalent σ-bonds raise expectations of exceptional mechanical strength.

Producing isolated chains of carbon atoms has remained a challenge for a long time, due to the difficulties in their synthesis and characterization. It is clear that only techniques allowing imaging with atomic resolution would give reliable information about the existence of carbon chains. A few years ago, the first electron microscopy images of carbon chains appeared [20–22]. This gave the field a new impetus; in particular since a new generation of transmission electron microscopes (TEM) with improved spatial resolution became available. Most recently, the integration of scanning tunneling microscopy tips into the specimen stages of TEMs allowed the first electrical characterization [23] and therefore a more detailed investigation of carbon chains. The study of the electrical conductivity has been motivated by measurements on chains of metal atoms that showed quantized conductance [24].

Carbon chains have been subject of numerous theoretical studies since more than a decade. Calculations are facilitated by the fact that chains consist of typically 10 atoms only and constitute very small systems as compared to two- or three-dimensional nanomaterials. Of high importance, however, is the nature of the contacts at the ends of the chains. The mechanical and electrical properties of carbon chains have been calculated long before they became accessible to experimental observation (quantitative experimental information about the mechanical properties is still lacking). However, several difficulties in the modeling of chains appeared that made their simulation quite laborious.

Bulk carbyne with its unclear stability and chemistry will not be discussed in detail in this review. Instead, we will focus on isolated chains of carbon atoms as the elementary building blocks of sp^1^-hybridized carbon. We will not address the interaction between different chains (although this interaction is important in a bulk solid) and, hence, study the interesting properties of linear arrangements of carbon atoms.

## Review

### The model of linear sp^1^-hybridized carbon

**Figure 1:**
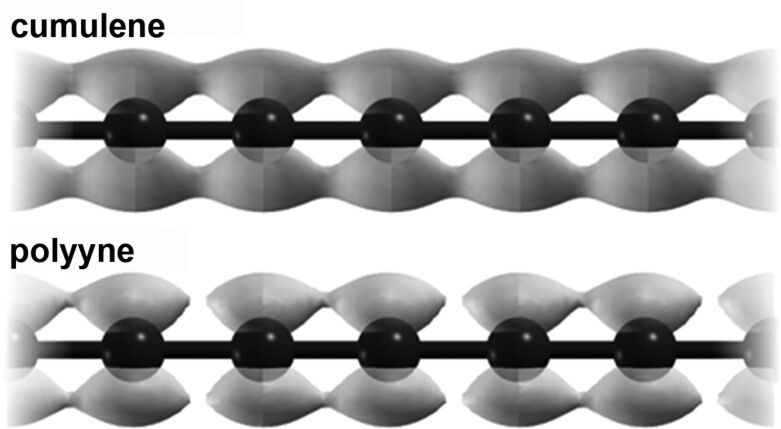
π-Electron distribution in cumulene (top) and polyyne (bottom). Calculation by A. Botello-Mendez and J.-C. Charlier. Reprinted with permission from [23]. Copyright 2013 American Chemical Society.

Linear sp^1^-hybridized carbon already results from the logical reduction of dimensions in carbon materials. Due to the similarity with the bonding characteristics in alkynes, in particular acetylene, the structure of sp^1^-hybridized carbon is addressed in many textbooks of chemistry although carbyne is not a standard phase of carbon like graphite or diamond. Two extreme cases for the electronic structure can be imagined, namely cumulene with double bonds throughout the chain (=C=C=C=C=) and polyyne with alternating single and triple bonds (-C≡C-C≡C-). Cumulene has a uniform distribution of the π-electrons along the chain, leading to metallic conductivity. In polyyne, however, the π-electrons are localized at the shorter bonds so that the continuous distribution is interrupted at each single bond (Figure 1).

Carbon chains are, as a one-dimensional system, susceptible to Peierls distortion [25]. According to Peierls’ theorem, the electron system of a perfectly one-dimensional crystal is unstable. The atomic periodicity is broken in such a way that the elementary period of the undistorted chain *a* is doubled to 2*a*. The calculated length of the double bond in cumulene is 1.282 Å; the single and triple bond lengths in polyyne are 1.265 Å and 1.301 Å, respectively [26]. The alternating shorter and longer bonds in polyyne result in an electronic bandgap. The gain in electronic energy due to the formation of a bandgap outweighs the increase in strain energy [27]. However, the gain in energy of the (unstrained) polyyne structure is only 70 meV/atom [28]. This is a small difference in comparison to the total cohesive energy of the order of 7.0 eV/atom in a chain.

Long-range interactions in the electron system of carbon chains determine their properties [26]. The π-electrons of carbon chains can be considered as a one-dimensional system of free electrons. If one atom in the chain experiences a small displacement, the local perturbation of charge leads to oscillations of charge density and interatomic forces, extending over longer distances along the chain. Such Friedel oscillations in one-dimensional systems decay as 1/*r*.

### Experimental observations of carbon chains

The first experimental indication for the existence of carbon chains has been deduced from a study of field emission from carbon nanotubes [29–30]. The emission characteristics of open tube ends were not in accordance with the expected emission from a perfect tube. It was suspected that an unraveling of the tubes had taken place so that the electrons were emitted from dangling chains of carbon atoms. Unexplained features in the electrical characteristics of graphene [31] or in the conductivity of breaking nanotubes [32] as well as in the Raman spectra from heat-treated carbon nanotubes [33] have been attributed to the formation of carbon chains. However, no independent proof for the presence of atomic chains has been obtained.

Early indications for the existence of chains have also been obtained from electron microscopy images of carbon nanotubes. Sometimes an unexplained fringe in the interior of a tube has been taken as an indication for the presence of an atomic wire located on the axis of a tube [34]. Much clearer indications, however, came from the observation of double-wall carbon nanotubes where the disintegration of the inner shell led to very short chains in the axial direction between the vanishing peapod-like residues [35]. Quite convincing pictures from carbon chains were also obtained when single-wall carbon nanotubes shrank under electron irradiation [20,36]. Just before the hourglass-shaped tube broke, a linear fringe, bridging the conical tube ends, appeared in the images. The studies have been extended recently and give some indications for the interaction between neighbouring chains [37]. Indications for the appearance of short segments of chains migrating on graphene layers have also been found [38].

**Figure 2:**
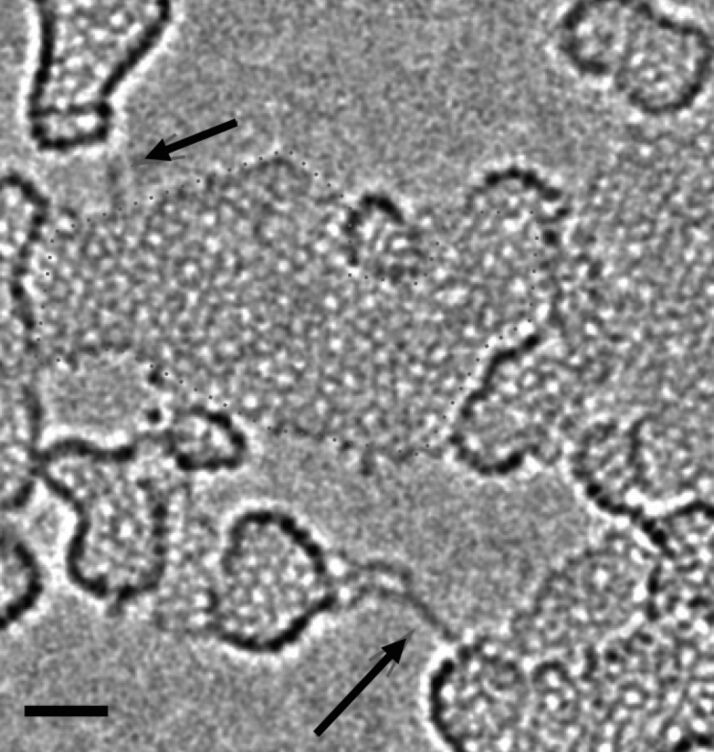
Electron microscopy image of carbon chains (arrowed). The carbon chains span between aggregates on top of a graphene layer (the contrast from the graphene lattice has been removed from the image by Fourier filtering). The scale bar is 1 nm. Courtesy of U. Kaiser, reprinted with permission from [22]. Copyright 2009 IOP publishing & Deutsche Physikalische Gesellschaft.

The first direct observations of the transformation from graphene to linear chains of carbon atoms have been reported in 2009 by Jin et al. [21] and Chuvilin et al. [22]. Both groups studied graphene monolayers by aberration-corrected high-resolution electron microscopy (Figure 2). The procedure has been quite simple: graphene membranes were irradiated with the electron beam that is used for imaging in the microscope. Sputtering of carbon atoms from the layers led to growing vacancies and holes. The phenomenon has been confirmed later [39–40]; in some experiments even two parallel chains were observed once a graphene ribbon has been thinned laterally to a certain minimum width. The narrowing graphene ribbons between two holes ended in atomic chains as the smallest possible bridges between neighbouring graphene areas. The chains had lifetimes of a few seconds under the electron beam until they ruptured. This was an unexpected observation, given the high stability of graphene. Why should not graphene ribbons just disintegrate or fold back to the neighboring graphene layers? This was the first indication that atomic chains are more stable than narrow graphene ribbons, at least under an electron beam.

Phase contrast images taken by TEM are often difficult to interpret; it is easy to mix up chains with graphene ribbons in side view. In this context, not all published images from carbon chains might be unambiguous. Despite some efforts, the atoms in chains have not been clearly resolved by TEM although aberration-corrected microscopes should have the necessary resolution power. Besides the contrast problem (single carbon atoms are rather weak electron scatterers), the vibration of the chain under the electron beam makes atomic resolution difficult. Therefore, the atoms in a chain could not be counted reliably until now; the measurement of the bond length and the question whether cumulene or polyyne has been observed is still unanswered although some indications have been given [37]. Since TEM only gives projections of the chains onto a screen and the chains may be curved or aligned randomly, not even the length of the chains could be measured with high accuracy.

**Figure 3:**
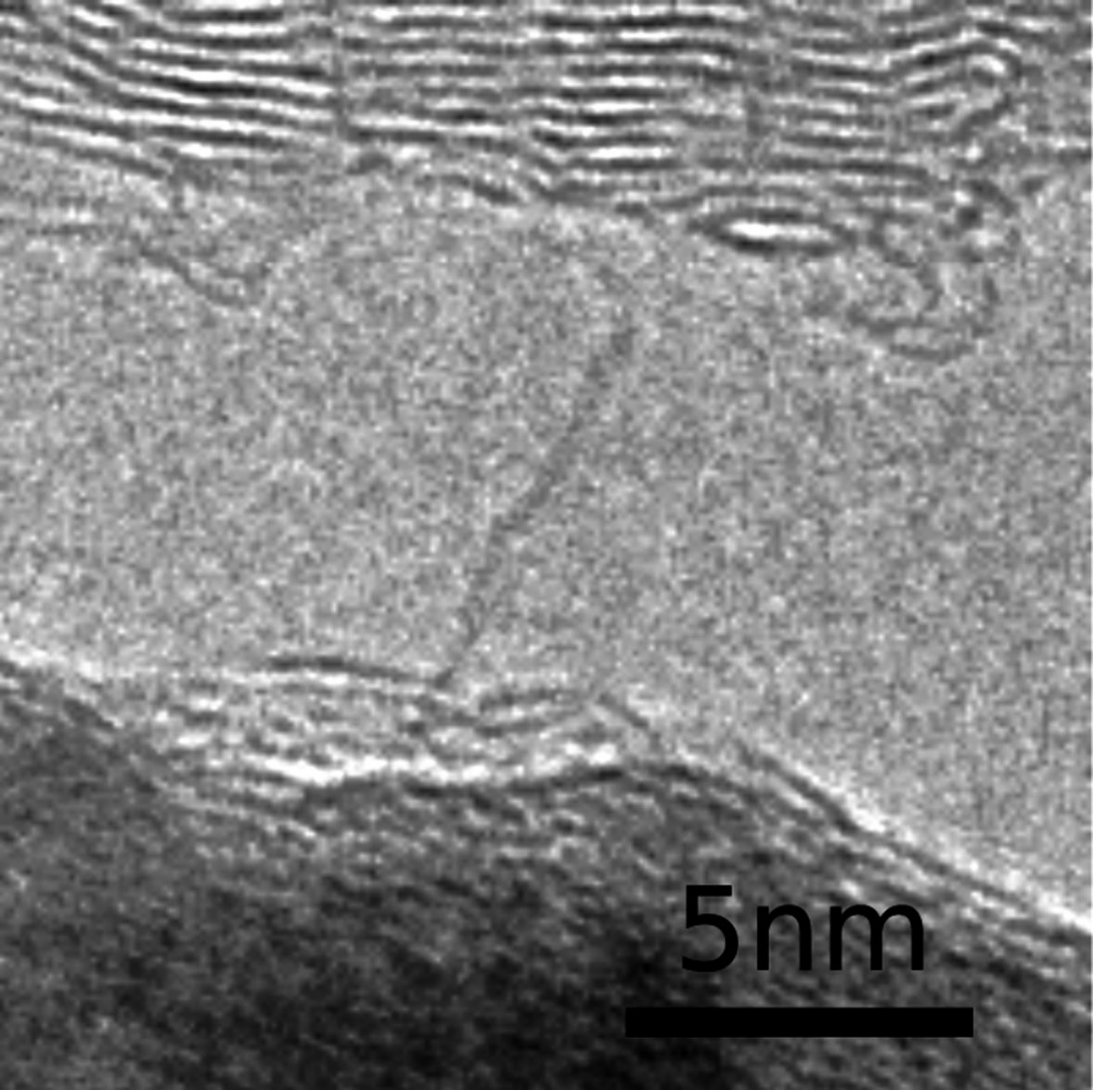
A carbon chain spanning between an iron crystal (bottom) and a graphitic aggregate (top). Both act as electrodes so that the electrical properties of the chain can be measured. Reprinted with permission from [23]. Copyright 2013 American Chemical Society.

Another electron microscopy experiment allowed not only the generation of carbon chains but also their electrical characterization [23]. By contacting a graphitic aggregate with a transition metal tip and passing an electrical current through the junction, carbon chains could be unraveled from the graphitic materials when the tip was retracted (Figure 3). The experiment was carried out under the electron beam in the microscope; therefore electron irradiation might have played a certain role during the formation of chains. Once the chains were created, current-voltage characteristics were taken. The conductivity of the chains was much lower than predicted from theoretical work. By applying a voltage of 1 V, currents of typically less than 10 nA were measured.

As mentioned above, Peierls distortion is an important effect that favours the electronic structure of polyyne. If tensile strain is induced by an external force, it is clear that the weaker single bonds will stretch by a larger amount than the stronger triple bonds. Therefore, strain has a considerable influence on the conductivity of chains [23,26,28,41]. Increasing strain leads to a decreasing π-electron density over the longer single bonds. This, in turn, hinders the electron transfer over the single bonds and opens the bandgap. Simulations show that a strain of 10% leads to a bandgap of 1.5–3.0 eV, depending on the approximation (DFT, GW) [19,23].

The electrical properties of chains can only be measured by contacts through single atoms. The bonding characteristics at this particular point are of paramount importance. One single bond will have a decisive influence on the transport. At the contact between a chain and a metal, the delocalization of the conduction electrons and their overlap with the π-electrons of the chain could lead to an ohmic contact with low resistivity. Contacts with graphene or other sp^2^ carbon structures, on the other hand, can be quite different, depending on a local sp^2^ or sp^3^ character at the junction. In the case of local sp^3^ bonding, the π-electron density would be low at the contact and make the electron transfer difficult.

**Figure 4:**
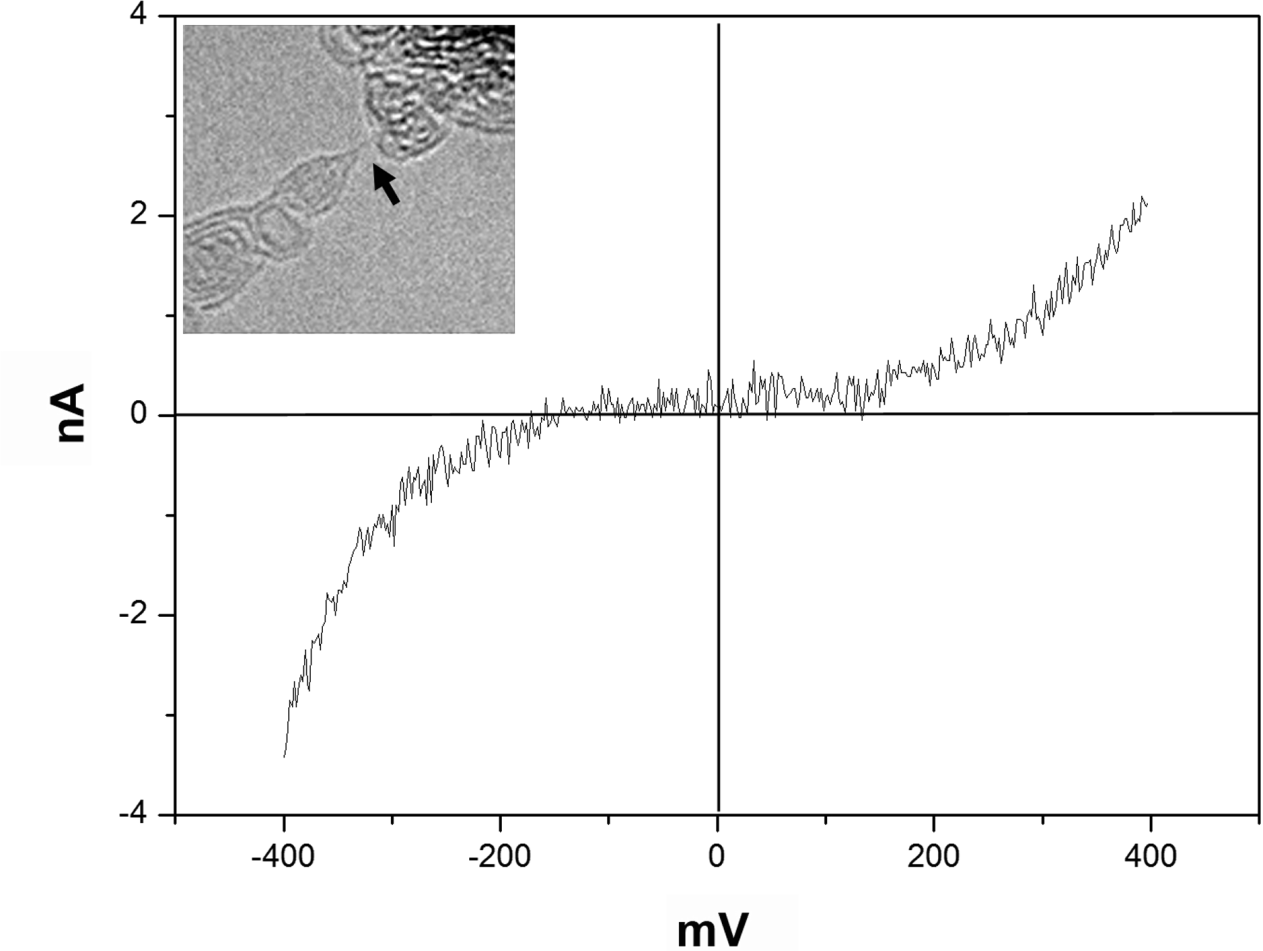
Measured current–voltage characteristic of a carbon chain (measurements by A. La Torre). A TEM image of the chain is shown in the inset.

The measured current–voltage characteristics show, for most chains, an S-type behaviour (Figure 4). This is characteristic of the existence of a bandgap. In view of the preferred configuration of polyyne and possible strain, the current–voltage curves are qualitatively understandable.

A conductance measurement on a related system has been carried out by STM [42]. Oligoynes functionalized with anchor groups have been contacted by an STM tip, in part in solution. However, it is unclear whether individual monoatomic chains without side-links to other molecules have been present between the anchors and served as conductivity-limiting molecular junctions.

The predicted outstanding mechanical properties of carbon chains still have to be confirmed experimentally. Preliminary results show qualitatively that chains have a high tensile stability against rupture. After the formation of chains between two contacts in the above-described procedure [23], the rupture of the chains after a few seconds of observation is often accompanied by a visible rebounding of the contacts. Since the system is under stress while one contact is retracted, the jumping of the contacts upon rupture of the chain is a convincing proof for a certain strength of the chains. If the elastic properties of the contacts were known or the forces could be measured, the ultimate strength of carbon chains could be determined quantitatively.

### Theoretical work on carbon chains

Due to the difficulties in the synthesis of these elusive atomic-scale objects, the last decade has only seen few experimental reports about carbon chains. On the other hand, there is no lack of theoretical work. Since carbon chains are typically just ten-atom systems, the computational effort seemed in the first instance to be moderate. However, several unexpected difficulties in conventional computation schemes such as density functional theory (DFT) occurred, leading to the application of more sophisticated and, accordingly, more computationally intensive techniques of simulation. While DFT gave correct calculations of lattice constants and energies, the bandgaps have been considerably underestimated. Many-body perturbation theory (MBPT) in the GW approximation including electron–electron interactions resulted in more accurate calculations. Without discussing the applicability and reliability of different computational techniques, a short overview of some predicted properties of carbon chains will be given in this chapter.

#### Electrical properties

Due to the continuous overlap of the π-electrons along the chain, cumulene is metallic. Two degenerate half-filled p_x_ and p_y_ bands cross the Fermi level of cumulene that shows a quantum ballistic conductance of 4e^2^/h. In polyyne, however, the unit cell is doubled by the difference in length of the alternating bonds. This leads to a bandgap of 0.32 eV at the edge of the Brillouin zone, lowering the total energy per atom by 2 meV. The electrical current flowing through a chain is expected to be axially symmetric with vanishing current on the axis where the non-conducting σ-electrons are localized. The conducting π-electrons carry the current with a maximum in a bulge at about 1 Å from the axis [19].

Although the chains should theoretically have a high conductivity, experiments showed much lower values than predicted. Yuzvinsky et al. reported conductivities one order of magnitude lower than expected [32], but in this preliminary study the occurrence of the chains was just assumed. Later experiments, where the chains were clearly visible [23,43], showed still lower conductivities. Current–voltage curves are rather simple two-terminal measurements that give access to the electrical characteristics of carbon chains. Different behaviours have been predicted, depending on the nature of the contacts and the number of atoms in the chain. Low-bias I–V curves should be linear whereas a negative differential resistance could occur at higher bias due to a shift of the conduction channels relative to the electronic states of the contact electrodes by the external bias [44–45]. The negative differential resistance has been proposed in order to explain an experimentally observed feature in the electrical characterization of breaking nanotubes [32].

Several computational studies predict that the electrical properties of chains with an even number of atoms are different from those with an odd number [46–49]. Generally, even-number chains should have a higher conductivity. In short chains, however, ballistic transport has only been predicted for chains with odd numbers of carbon atoms [49]. By considering cumulene chains between two metal electrodes, Lang and Avouris [46] have found that even-number chains have a lower density of states (DOS) at the Fermi level than odd-number chains. In free chains (no contacts), the highest occupied molecular orbital (HOMO) is half occupied if the chain has an even number and full occupied for an odd number of atoms. When the chains have metal contacts at their ends, however, the HOMO level of even chains is essentially full, leading to a low DOS at the Fermi energy whereas odd chains have a partially filled HOMO level. A high DOS at the Fermi level leads to a high conductance. Song et al. [48] have calculated the influence of even/odd number of atoms on the I–V characteristics of chains. Plateaus are found where the current becomes independent of bias for even chains whereas in odd chains the current increases monotonically with bias. Even chains should have a better conductivity at low bias whereas odd chains conduct more at high bias. The dimerization resulting from the Peierls distortion is assumed to be responsible for this behavior.

**Figure 5:**
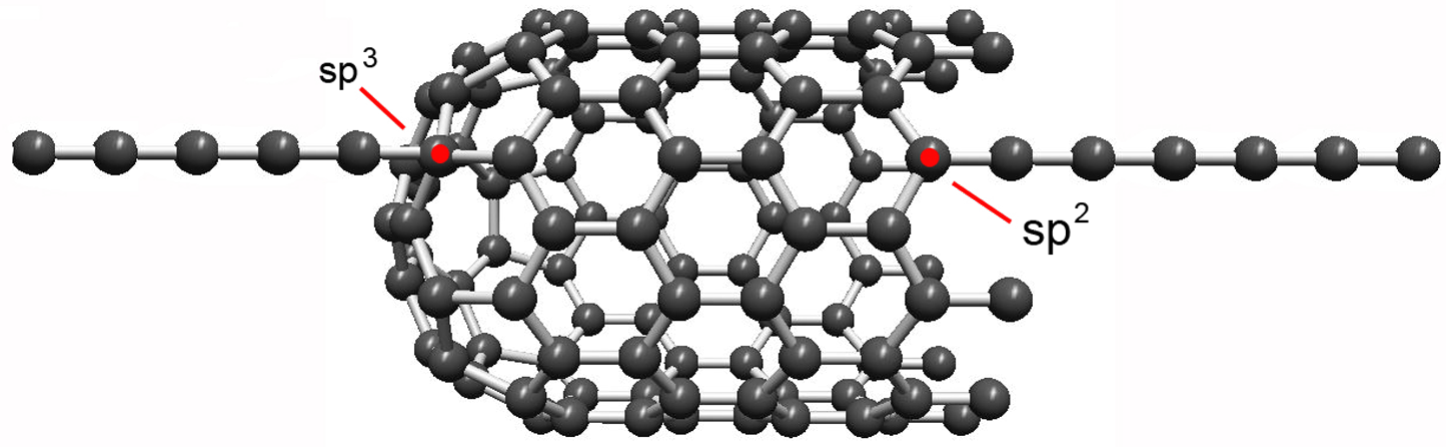
Different hybridization states of the end atoms of carbon chains when they are connected to a graphitic contact. The contact atoms are marked in red. As an example, chains are attached to the closed end of a carbon nanotube (left), leading to local sp^3^-hybridization, and to an open end where the contact atom is sp^2^-hybridized (right).

It is obvious that the nature of the contacts has a high influence on the electrical behavior of the system. The conductivity of a one-dimensional π-electron system with ideal contacts (transmission *T* = 1) would be 4e^2^/h. However, when the contacts are made of graphene, the characteristics are different from metal-chain systems. The transport at the contacts occurs through narrow resonant states in the chain, due to reflections at the interface to the contacts [50]. Close to the Fermi energy of perfect graphene, the transmission *T* decreases linearly with electron energy. The low transmission results from the vanishing of the DOS in undoped graphene. The reflection is high if the density of states in the contacting material at low energy is small. Another aspect is the local hybridization of the carbon atoms at the contact. A sp^3^-hybridized carbon atom (e.g., when the chain is connected to the middle of a graphenic sheet or the wall of a carbon nanotube as shown in Figure 5) leads to a missing π-electron and, accordingly, to a reduced charge transfer. On the other hand, when the chain is attached to a graphene edge, the contact atom is sp^2^-hybridized, allowing a continuous π-electron system over the contact and better conductivity. Therefore, the choice of sp^3^ or sp^2^ termination would allow switching the chain on and off [51].

The contact can also be considered in the context of doping since electrons can be transferred from the electrodes to the chain (charge transfer doping) [47], thus increasing the carrier density. The Fermi level of metallic contacts would be aligned to the LUMO of the carbon chain. Localized charge at the metal-chain contacts would also generate Schottky barriers. In contrast to conventional semiconductors, doping by introducing foreign atoms (donors or acceptors) into the chain would not be appropriate since these important perturbations would lead to electron localization.

It is well known that the electrical properties of graphene depend strongly on adsorbed species that might even act as dopants. A similar influence of attached atoms or molecules might occur in carbon chains since their conductivity is also carried by the π-electron system. The influence of imperfections such as adsorbed impurities on the electrical properties of chains has been studied by Shen et al. [49]. If ballistic conductance occurs, adsorbed species such as hydrogen should have no influence. Oxygen impurities, on the other hand, are predicted to lower the conductivity of chains. Of particular experimental importance but not studied in detail yet would be the conductivity of a chain that is lying on a substrate.

For possible applications in devices, a rectifying behavior of the contact would be of interest. It has been suggested that changing the number of atoms by one (switching between odd and even number of atoms) can change the direction of rectification [52]. On the other hand, asymmetric contacts, e.g., different metals or a metal and a carbon contact at the two ends of the chain, should lead to rectification. It has also been proposed that switching might be possible when chains are terminated by five-membered carbon rings and exposed to strain [40].

Of further interest is spin transport that has been predicted by Zanolli et al. for carbon chains with covalent bonds to graphene ribbons [53]. Chains with odd number of atoms should carry such a spin polarization whether they are metallic or semiconducting (depending on the edge of the graphene ribbon at the contact). Hence, carbon chains connected to graphene ribbons could be used as spin polarized semiconductors. While Zanolli et al. did not obtain a magnetic moment on even-number chains, a triplet state appeared in even-number chains upon twisting by 90 degrees [28]. It has already been predicted in 1972 that twisted odd- or even-number chains have a triplet ground state [54].

#### Mechanical properties

Motivated by the outstanding mechanical properties of graphene, the response of carbon chains to stress, bending, or torsion has been addressed in several theoretical studies. Quantitative experimental information, however, is still lacking. The ultimate tensile strength (fracture) of a chain has been predicted to be of the order 10^−8^ N [19,55] whereas it is approximately 40 N/m in graphene [56]. Liu et al. [19] have related the fracture strength to a specific strength of carbon chains of 6.0–7.5 × 10^7^ Nm/kg which would be the highest of all known materials.

A Young’s modulus of 3.3 × 10^13^ Pa has been calculated [19] which is much higher than for graphene (approx. 10^12^ Pa). In sp^2^ networks like graphene or carbon nanotubes, the hexagons are able to elongate in the direction of strain and contract in the normal direction. In that case, the bond angles change but not the bond lengths. In carbon chains, however, the bonds are loaded along their alignment and strained which needs a much higher force. Carbon chains could thus be considered as the stiffest known material. This is supported by the specific stiffness, which for carbyne is predicted to be 10^9^ Nm/kg [19], clearly larger than for graphene (4.5 × 10^8^ Nm/kg [56]) or diamond (3.5 × 10^8^ Nm/kg [57]). The ultimate tensile strength corresponds to an ultimate strain of the order of 15% (graphene can be strained up to 20%). Liu et al. have also calculated a bending stiffness *K*= 3.56 eV·Å. This can be related to a persistence length *l*_p_ = *K*/*k*_B_*T* = 14 nm (corresponding to a chain of 110 atoms) at *T* = 300 K which is comparable to many polymers. Taking into account that different values for the thickness of carbon chains have been assumed, quite similar values have been found by other authors [58–59].

The high ultimate strength of carbon chains is reflected in the simulation of fracture of graphene-like networks under mechanical stress. Before a graphene ribbon breaks, carbon chains appear at the breaking edge as the ultimate junctions before rupture [60]. Even the behavior under twist has been calculated [19,51]. However, this is only applicable when rotationally non-symmetric groups are added to the ends of the chain (a chain is perfectly symmetric under rotation so that a twist is not defined). The chains can, for example, be stabilized by sp^3^ or sp^2^ end groups; the latter are sensitive to torsional strain [51].

Chains should also disintegrate in electric fields exceeding 4 × 10^10^ V/m [30]. This has already been discussed in the 1990s in the context of field emission from carbon nanotubes and the possible unraveling of carbon chains from their ends [29].

#### Chemical stability

The first question concerning the stability of chains is their energy in comparison to other carbon allotropes. It has been shown by calculations that carbon chains are energetically lower (more favourable) than extremely thin single-wall carbon nanotubes [5,61]. Indeed, no nanotubes with diameters below 4 Å have ever been observed. It has been shown by DFT calculations that an infinite chain is more favourable than an armchair (3,0) or zigzag (2,2) single-wall carbon nanotube [61]. The smallest experimentally identified carbon nanotube is the (3,3) nanotube with a diameter of 4 Å. The instability of extremely thin nanotubes is due to the increasing pyramidalization angle in small sp^2^ structures, leading to an increasing sp^3^ character of the bonds. The stability in comparison with narrow graphene ribbons will be discussed below in the context of defects and irradiation.

One of the most discussed properties of sp^1^-hybridized carbon is its chemical stability. Two parallel chains develop cross-links; this is an exothermic reaction with an activation barrier of 0.6 eV. In the lowest energy configuration the chains would have cross-links every 2.2 nm (17 atoms) [19]. The formation of such cross-links, where local sp^2^ hybridization occurs, is considered as the mechanism of degradation of carbyne [62].

An important question is whether isolated chains have a maximum length above which they become unstable. By electron microscopy observation, lengths up to 15–20 atoms have been found [21,23]. In other experiments, the synthesis of chains with lengths up to 44 atoms has been claimed [8]. It has become clear that the end termination has an important influence on the length of the chains. Linear chains of up to 28 atoms have been stabilized by nonreactive groups at the ends [10]. Hydrogen-terminated polyyne chains with lengths up to 20 atoms have already been synthesized [11,63]. The synthesis of the less stable cumulene appeared to be more difficult. Only short chains with two H atoms at each end have been found [64–65]. In a most recent study, carbon chains terminated with Pt atoms at their ends have been observed [66]. Chains might close their ends by forming rings. These have been proposed in several studies [67–69] but never seen. Carbon rings might be the ground state for carbon clusters with less than 20 atoms or precursors for the formation of more complex structures such as carbon nanotubes or fullerenes [18]. The rings might also consist of equal aromatic or alternated antiaromatic bonds.

Of current interest are also chemically related linear modifications, e.g., chains of B and N atoms that might have an even higher cohesive energy per unit cell than carbon chains [70]. A recent electron microscopy study shows the first observation of BN chains that formed under electron irradiation of BN layers [71]. These chains are predicted to be insulating with a bandgap comparable to two-dimensional BN. The Peierls theorem is therefore not applicable and no bond alternation is expected.

#### Formation and behaviour under electron irradiation

Carbon chains appear in the disintegration of both carbon nanotubes and graphene under electron irradiation. Apparently, the sputtering of carbon atoms from sp^2^ materials favours the transformation to the sp^1^ phase under certain conditions. Carbon atoms are displaced when the electron energy exceeds a certain threshold [72–74]. For perfect (infinite) graphene, the threshold energy of the electrons is around 80 keV [75]. Undercoordinated edge atoms can be sputtered off at lower energies although the edges tend to reconstruct [76]. The same holds for atoms next to unreconstructed vacancies. As for graphene, the displacement threshold for carbon chains should be highly asymmetric, being much higher on the axis than normal to it. Accordingly, atoms should be displaced normal to the chain. For carbon chains, the values have not been determined yet, neither experimentally nor theoretically. First experimental indications are the lifetimes of chains under the electron beam. Under typical conditions for electron microscopy observations (electron energy 200 keV, beam current density 10 A/cm^2^), a displacement rate of 0.1/s is obtained under the assumption that the lifetime of the chains is limited by irradiation [23]. The ends of the chains are contacted by graphene or metals in this case.

**Figure 6:**
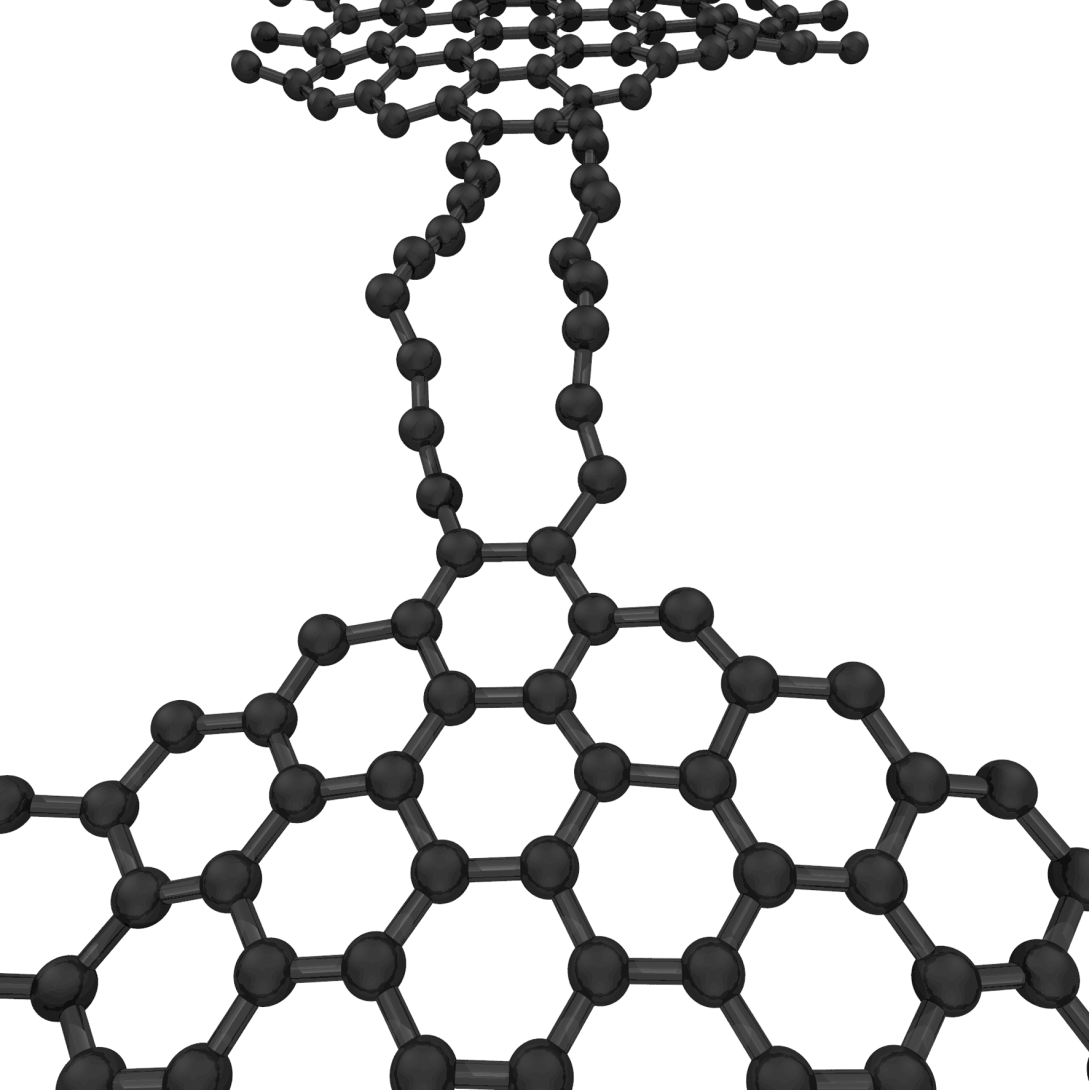
Formation of a pair of carbon chains during the breakage of a graphene ribbon under electron irradiation. The calculation shows the effect of continuous atom removal from a graphene sheet. Courtesy of J. Kotakoski.

The formation of chains under irradiation of graphene indicates that either the chains are energetically more stable than narrow graphene ribbons (resp. extremely thin tubes) [61] or that the atom displacement rate is smaller in chains. Jin et al. considered a ribbon that is thinned by the electron beam down to a line of hexagons that can be considered as two interlinked chains [21]. The calculated formation energy of a zigzag-terminated single-hexagon ribbon is 1.22 eV/Å which is higher than that of a carbon chain (0.76 eV/Å). The splitting of such a narrow graphene ribbon into two isolated chains would therefore be energetically favorable. The simulation of the breaking of graphene ribbons under irradiation shows the appearance of chains, bridging the gap between the separating ribbons for a short time (Figure 6) [76]. This occurs although the separation of a narrow graphene ribbon into two chains requires a high energy (18 eV/atom). A similar picture has been obtained in calculations of the fracture of strained graphene ribbons at grain boundaries [77]. Chains often appear as the last link between two separating grains. The kinetics of disintegration is also of importance as shown by Kotakoski et al. [76]. Carbon atoms from zigzag edges at a graphene ribbon are sputtered much faster than from armchair edges; the latter having almost the same stability under irradiation as bulk graphene. Exposing a square sheet of graphene to irradiation would lead to shrinkage from the zigzag edges until a chain of atoms is left.

## Conclusion

The existence of sp^1^-hybridized carbon has been secured experimentally and is undisputed. The high instability of bulk carbyne, however, due to cross-linking between chains of carbon atoms, made the availability of this material in bulk quantities and under ambient conditions very unlikely. The research concentrates therefore on isolated chains. Their outstanding mechanical and electronic properties, as theoretical work predicts, are still waiting for experimental confirmation. Preliminary experiments [23] do not confirm the predicted electrical conductivity. No experimental determination of the mechanical properties has been undertaken yet. The difficulty in all experiments is the production and handling of these atomic-size objects that appear to be highly reactive. Therefore, many questions remain open and need to be answered. It is not even clear if isolated chains are inherently unstable, e.g., above a certain length. It is clear, however, that the termination of the chains is crucial. End contacts transfer charge to the chains and determine the ultimate mechanical strength. Open chains react immediately with their environment or close by forming rings. Experimental efforts to synthesize longer chains have to be undertaken. The influence of end contacts as well as the stability of chains with a side contact, e.g., attached on a substrate, needs to be clarified.

In view of the predicted properties of carbon chains, the question arises if we can ever make use of their outstanding specifications. In many applications, bulk materials containing a large quantity of long chains would be needed. However, in nanoelectronic devices, working with single molecules or few-atom clusters, isolated chains of carbon atoms could find their applications in the future. In some respect, they might be superior to graphene ribbons whose properties depend strongly on the saturation of the edges. As we have seen from different electron microscopy studies, carbon chains are more stable than extremely narrow graphene ribbons. It could therefore be speculated that, once graphene comes to its limits, chains of carbon atoms become the building blocks in the ultimate miniaturization of carbon electronics. But even if this will never come, carbon chains will remain of interest as the textbook case of an extreme hybridization of the carbon atom.
